# Can an insole for obese individuals maintain the arch of the foot against repeated hyper loading?

**DOI:** 10.1186/s12891-019-2819-2

**Published:** 2019-10-11

**Authors:** Yuki Saito, Takako S. Chikenji, Yuichi Takata, Tomoaki Kamiya, Eiichi Uchiyama

**Affiliations:** 1 Graduate School of Health Sciences, Sapporo Medical University, South 1 West 17, Chuo-ku, Sapporo, 0608556 Japan; 2 Graduate School of Health Sciences, Hokkaido University, Sapporo, Japan; 3 Department of Orthopaedic Surgery, Sapporo Medical University, Sapporo, Japan

**Keywords:** Adult acquired flatfoot deformity, Insole, Obesity, Cyclic load, Bony arch index

## Abstract

**Background:**

Insoles are often applied as preventive therapy of flatfoot deformity, but the therapeutic effects on obese individuals are still controversial. We aimed to investigate the effect of insole use on time-dependent changes in the foot arch during a repeated-loading simulation designed to represent 20,000 contiguous steps in individuals with a BMI value in the range of 30–40 kg/m^2^.

**Methods:**

Eighteen cadaveric feet were randomly divided into the following three groups: normal, obese, and insole. Ten thousand cyclic loadings of 500 N (normal group) or 1000 N (obese and insole groups) were applied to the feet. We measured time-dependent change in arch height and calculated the bony arch index (BAI), arch flexibility, and energy absorption.

**Results:**

The normal group maintained more than 0.21 BAI, which is the diagnostic criterion for a normal arch, throughout the 10,000 cycles; however, BAI was less than 0.21 at 1000 cycles in the obese group (mean, 0.203; 95% confidence interval [CI] 0.196–0.209) and at 6000 cycles in the insole group (mean, 0.200; 95% CI, 0.191–0.209). Although there was a significant time-dependent decrease in flexibility and energy absorption in both the obese and insole groups (*P* < 0.001), the difference between 1 and 10,000 cycles were significantly smaller in the insole group than in the obese group (*P* = 0.024).

**Conclusions:**

Use of insoles for obese individuals may help to slow time-dependent foot structural changes. However, the effect was not enough to maintain the foot structure against repeated hyper loadings.

## Background

Adult acquired flatfoot deformity (AAFD) is a foot disorder characterized by collapse of the medial longitudinal arch, forefoot abduction, and hindfoot eversion [1]. This foot deformity is associated with gradual loss of function with intense pain, and causes gait disorder in activities of daily living (ADL) [2]. Although the etiology of AAFD remains unclear, a high body mass index (BMI) value is believed to be a risk factor for AAFD in both men and women [3].

Insoles are often used as the first-line conservative treatment for AAFD because of their immediate availability, lower cost, and ease-of-use by the patient [2, 4, 5]. The kinematic and therapeutic effects of insoles have been reported in AAFD patients [6, 7], and some kinematic studies have demonstrated that insoles reduce abnormal motions, such as eversion of the forefoot and subtalar joint under single loading in AAFD [8, 9]. However, the effects of insoles on AAFD with obesity remain controversial [6, 10] because these abnormal motions worsen with increasing axial load [11, 12]. One follow-up study has reported that 62.5% of patients with AAFD in whom conservative treatment failed had a BMI > 30 kg/m^2^ [4]. These results suggest that the efficacy of insoles may be limited by patient characteristics, and time could modify the effect of insoles for obese individuals.

The purpose of this study was to investigate the effects of insole use on time-dependent changes in the arch structure of cadaveric feet during repeated loading. Our experimental model was designed to represent 20,000 contiguous steps (15–18 km walking) in individuals with BMIs of 30–40 kg/m^2^. We asked, (1) can the insole maintain the foot arch against repeated loading in the presence of obesity and (2) does the use of an insole affect the flexibility and energy absorption of the foot arch in obesity?

## Methods

### Specimen preparation

This study was approved by our institutional review board (IRB). All specimens were obtained from the body donation program run by the Sapporo Medical University (Shiragiku-kai). This program donates their bodies for medical education and research after death. The donation was agreed to by the members while they are alive. We studied 18 fresh frozen cadaveric feet from 4 women and 14 men with a mean age of 82 years (range, 59–93). Fourteen specimens were left feet and 4 were right. Cadaveric feet with deformities, including joint contracture, postoperative scarring, and low or high arch as evaluated by the bony arch index (BAI) [13], were excluded from this study. The specimens were randomly divided into the following three groups: normal (*n* = 6), obese (n = 6), and insole (n = 6).

Each specimen was cut at the proximal third of the leg. Soft tissues were removed 5 cm from the cut ends of the tibia and fibula. The tibia and fibula were fixed with 2-mm Kirschner wires and embedded in poly(methyl methacrylate) with a 5-cm-diameter acrylic tube. The posterior tibial tendon was exposed 5 cm proximal from the medial malleolus, and the navicular tuberosity was exposed with a minimal skin incision.

**Fig. 1:**
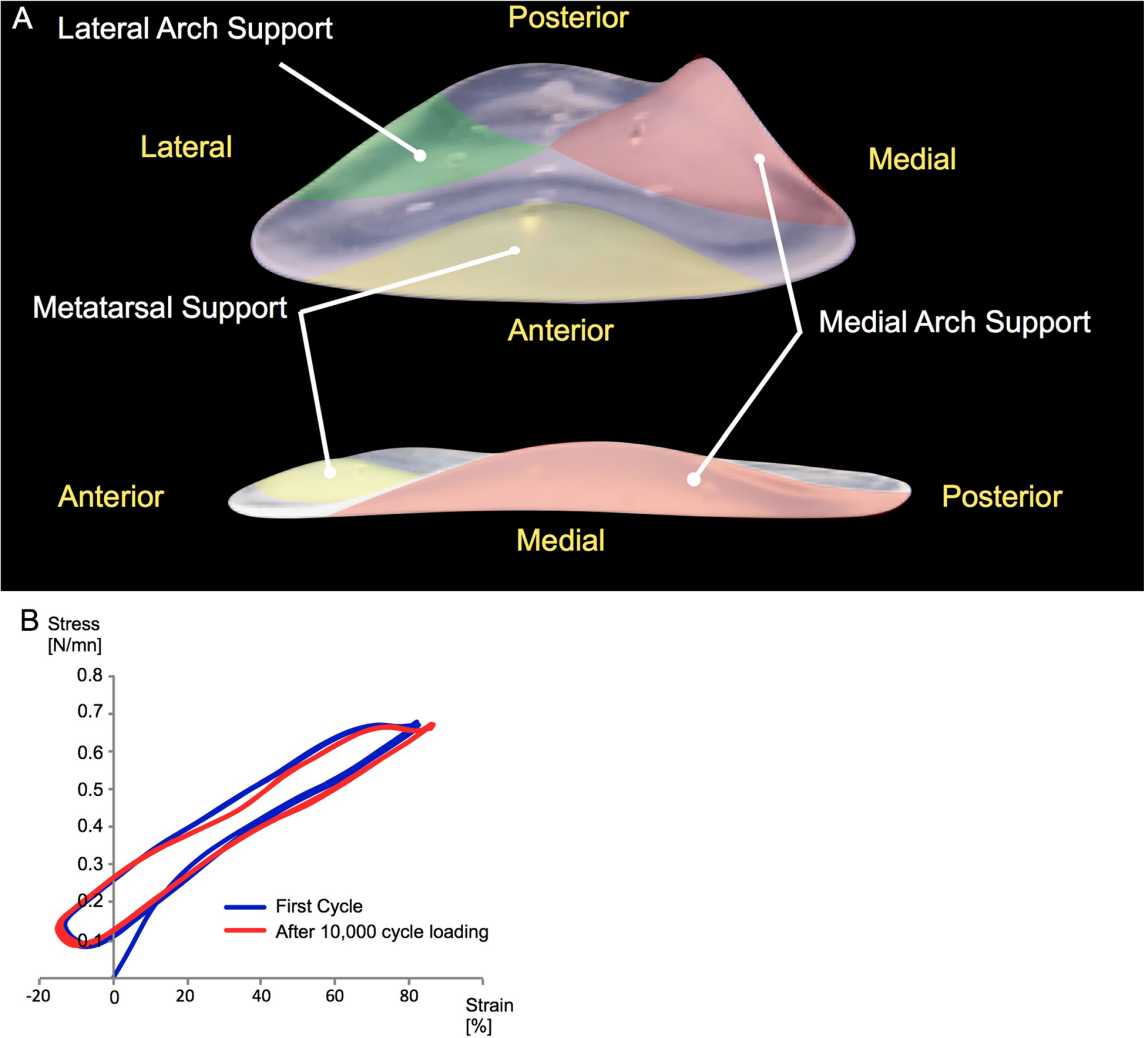
Anterior and medial view of the silicone rubber insole. Silicone rubber insole was designed to support the metatarsal arch and medial and lateral longitudinal arch of the foot (**a**). Stress-Strain curve of silicone rubber. The blue line indicated stress-strain curve in the first cycle and the red line indicated stress-strain curve after 10,000 cycle loading (**b**)

The foot was set on a force plate with the tibial shaft perpendicular to the plate, and then fixed on a custom jig at the neutral position. The normal-weight condition was simulated by applying a 500-N axial load to the normal group specimens. Obesity was simulated by applying a 1000-N axial load (representing class I-II obesity, BMI range 30–40) to the obese group and insole group specimens. The insole (arch support, Nakamura Brace Co., Shimane, Japan) was designed to support the medial and lateral longitudinal transverse metatarsal arch of the foot (Fig. 1a). The insole was made of silicone rubber, which has a low permanent compression set and keeps its original shape and mechanical property after a fatigue test with 10,000 loading cycles (Fig. 1b).

### Experimental system

**Fig. 2:**
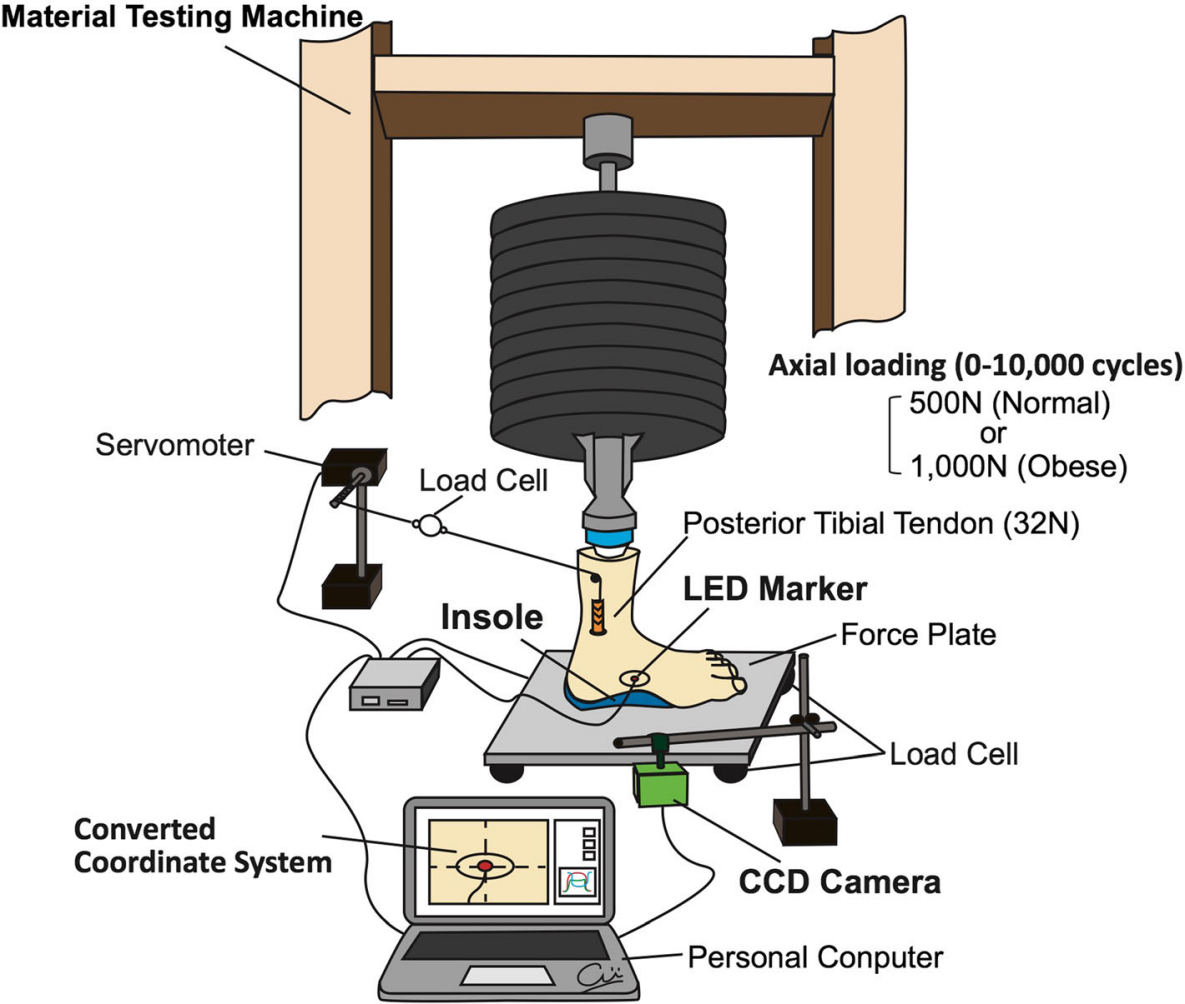
Diagram of experimental system. The foot, with or without insole, was set on a force plate. Ten thousand cyclic axial loadings (normal group, 500 N; obese and insole groups, 1000 N) were applied to the proximal end of the leg. The posterior tibial tendon was pulled proximally (32 N) by a servomotor during the weight-bearing phase of cyclic loading. LED displacement was monitored via a CCD camera, and the image was converted to a coordinate system

The manufactured experimental system, which simulates the midstance phase of gait, has been described in detail [14]. Loads were applied to the proximal end of the specimens using a material testing machine (AG-I, Shimadzu, Kyoto, Japan). The load was set at 500 N (normal weight) or 1000 N (obesity), and was monitored by four load cells (FC22, Measurement Specialties Inc., Hampton, VA, USA; capacity 45 kgf, nonlinearity ±1% full scale) under the force plate (Fig. 2). The cyclic axial loading system, which was composed of the material testing machine and a 500-N or 1000-N weight hung on the upper cross-head, oscillated at a cross-head speed of 10 mm/s and performed up to 10,000 cycles. We used a personal computer with control software (Trapezium, Shimadzu, Kyoto, Japan) to configure the cyclic loading program to achieve loads of 0 to 500 N or 1000 N. The frequency of loading was 1 cycle per second (1 Hz), and one cycle consisted of a weight-bearing phase (defined as at least 50 N of force applied to the foot) and a non-weight-bearing phase (defined as less than 50 N).

Time-dependent change in arch height during cyclic loading was monitored with a 2-dimensional analyzer, which consisted of a light-emitting diode (LED; Panasonic Co., Osaka, Japan) and a charge-coupled device (CCD) camera (Medisens Inc., Saitama, Japan; Fig. 2). A 1.6-mm × 0.8-mm rectangular LED was attached to the tuberosity of the navicular bone, and displacement of the LED light was monitored via a 640 × 480 pixel resolution CCD camera connected to a personal computer. The image was converted to a 2-dimensional coordinate system, and navicular height was calculated from the y-axis displacement of the LED. The y-axis was located along the tibial shaft, and the x-axis was parallel to the horizontal plane of the force plate. The translational accuracy of this program was 0.06 mm (0.2% full scale).

Muscle activity during the midstance phase of gait was replicated by exerting traction on the posterior tibial tendon, whose activation is essential for maintaining the foot arch in normal-weight conditions [14]. The posterior tibial tendon was exposed with a minimal skin incision 5 cm proximal from the medial malleolus, and towed by a servomotor (Medisens Inc., Saitama, Japan; maximal torque 6 kg·cm, rotational speed 600 degrees/s) to simulate contraction of the tibialis posterior muscles. The traction force was set at 32 N, which was estimated from the physiological cross-sectional area and electromyographic studies [15, 16]. Once an axial load of 150 N had been applied to the foot, traction force was applied to the tibialis posterior tendon and monitored using a load cell attached to the servomotor (accuracy ±0.01 N).

The LED displacement data, the amount of load applied to the foot, and the traction force on the tibialis posterior were sampled at 20 Hz and analyzed using a commercial software (Medisens Inc., Saitama, Japan).

### Data analysis

The arch structure was evaluated using the BAI [13]; this was calculated according to the equation BAI = h/l, where h is the navicular height and l is the foot length. Foot length was measured from the calcaneus tubercle to the first metatarsophalangeal joint, and navicular height was measured from the coordinate y-axis. BAI less than 0.21 and from 0.21 to 0.27 during weight bearing was defined as low arch and normal arch, respectively. Flexibility and energy absorption were also calculated to assess dynamic characteristics of the arches. Flexibility (μm/N) was assessed via deformation, which was calculated as the difference between weight-bearing and non-weight-bearing arch height divided by the load. Energy absorption (J) was calculated by multiplying the deformation by the load, then dividing by two [17]. Polynominal approximation was used for calculatiotn of an inflection point of time-dependent change of BAI. The polynomial approximation was used for calculating an inflection point of time-dependent change of BAI. In order to determine the best fit polynomial approximation, we used Akaike’s Information Criterion (AIC) [*AIC* = *n*{*log*(2*πS*_*e*_/*n*) + 1} + 2(*p* + 1)]. In this model, we determined that the 3rd order fit was the optimal approximation (R^2^ = 0.947; 95% CI, 0.932–0.963). The inflection point (*χ*_0_) was determined with a second derivative test [*f*^′′^(*χ*_0_) = 0] to determine the point of second to third deformity stage in fatigue behavior [18].

### Statistical analysis

Sample size was calculated on the basis of our preliminary data. A sample size of 6 specimens in each group was found to provide 80% power to detect difference in BAI. Results were expressed as mean and 95% confidence interval (95% CI). Normality was assessed using the Kolmogorov-Smirnov test, and we confirmed that all the data showed non-normal distribution. We conducted a Kruskal-Wallis test to assess overall differences among BAI, flexibility, and energy absorption at 1 cycle, and the difference between 1 and 10,000 cycles in terms of BAI, flexibility, and energy absorption among the three groups. Pairwise comparisons were made only when the Kruskal-Wallis test indicated statistical significance. *P*-values for multiple comparisons were adjusted by Bonferroni methods. A Friedman test was conducted to assess time-dependent change in BAI, flexibility, and energy absorption. Statistical analyses were performed using EZR, which is a graphical user interface for R (The R Foundation for Statistical Computing, Vienna, Austria) [19]. Two-sided *P*-values less than 0.05 were considered statically significant.

## Results

**Fig. 3:**
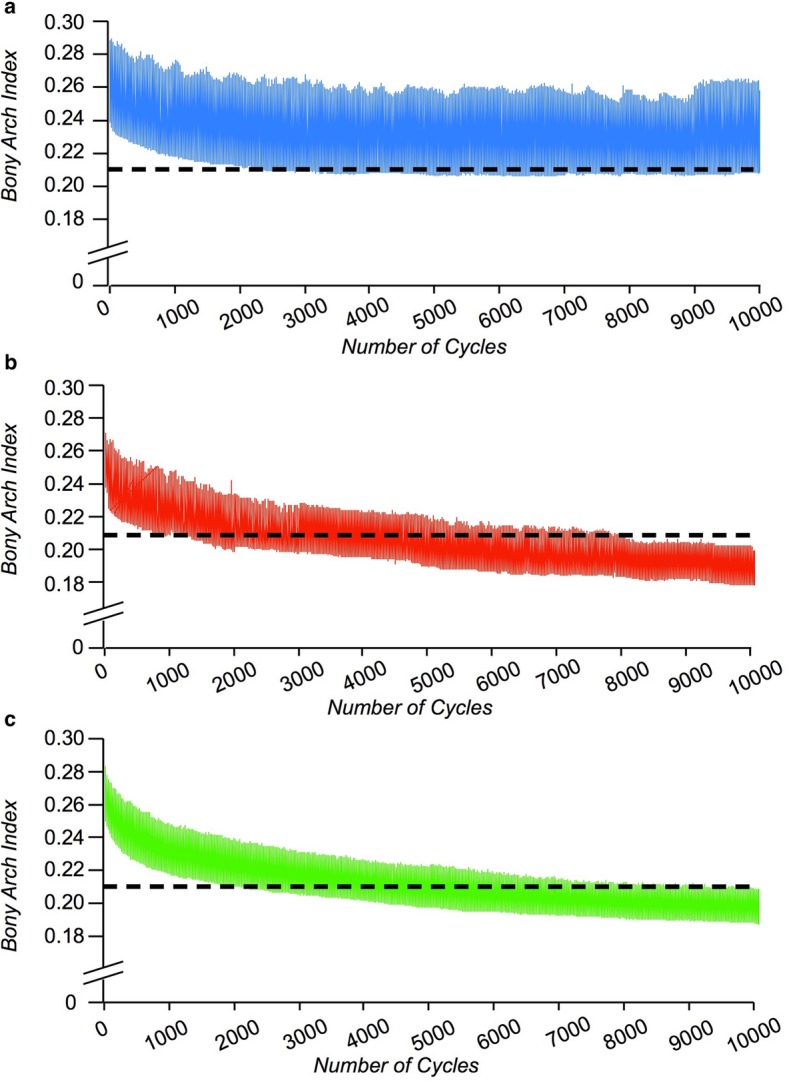
Typical change in BAI over time during cyclic loading. Typical change in BAI over time during cyclic loading in the normal (**a**), obese (**b**), and insole (**c**) groups. BAIs are plotted at the points of maximum and minimum load applied to the foot for 10,000 cycles. The dashed line indicates the diagnostic criterion for low arch (BAI less than 0.21)

Fig. 3 shows typical time-dependent changes in BAI in the normal, obese, and insole groups during the 10,000 cycle load and unload. The normal group maintains a BAI > 0.21—the diagnostic criterion for a normal arch—throughout the 10,000 cycles even in the non-weight-bearing phase; however, the BAI of the obese group dropped to < 0.21 even in the non-weight-bearing phase. The insole group maintained a BAI of around 0.21 in the non-weight-bearing phase.

**Fig. 4:**
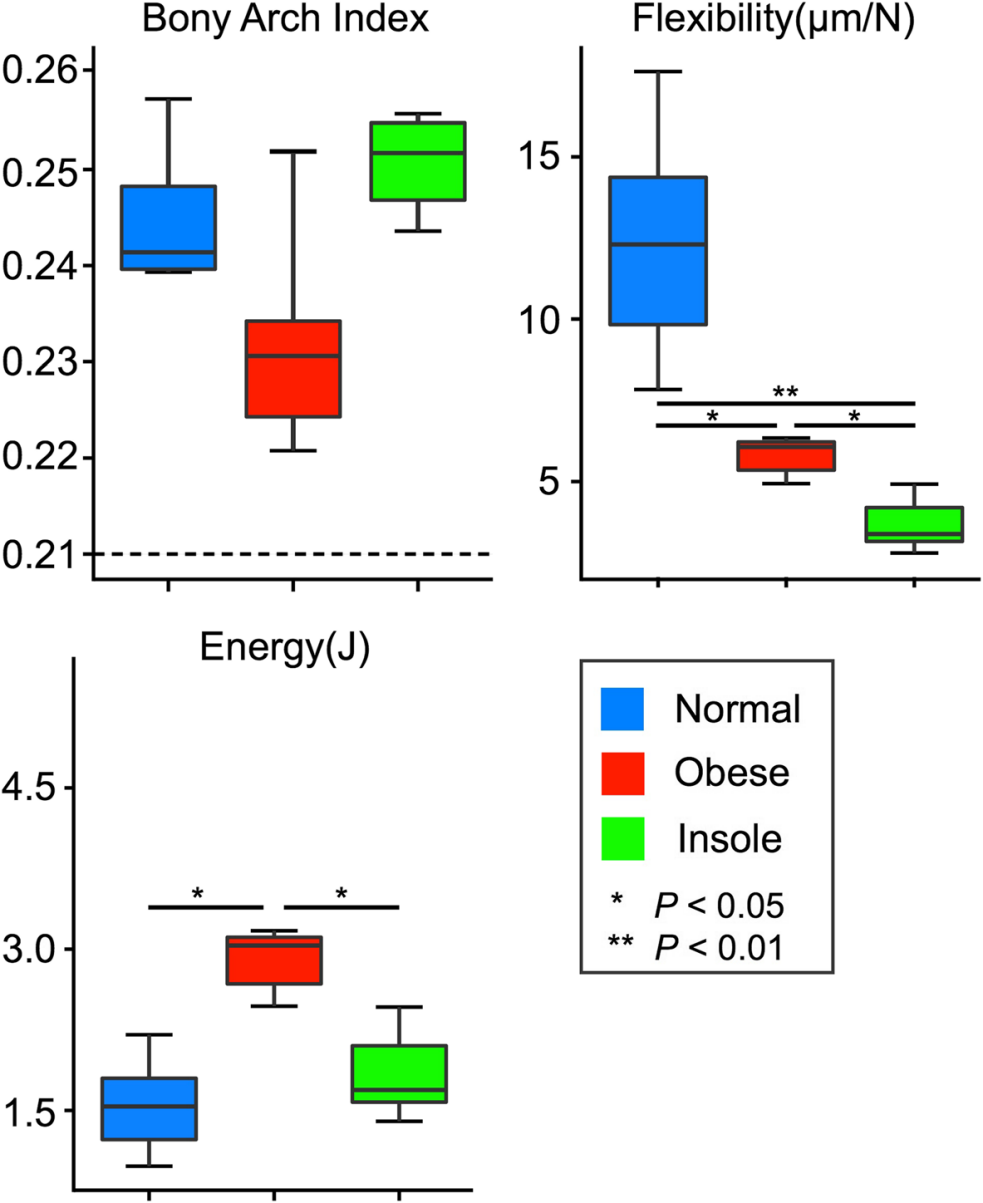
BAI, flexibility, and energy absorption at the first axial load. BAI, flexibility, and energy absorption at the first axial load in the normal, obese, and insole groups. Boxes represent the interquartile range (IQR), lines within boxes represent the median, and lines outside boxes represent 1.5 times the IQR

Although BAI at the first axial load was not significantly different between the three groups (Fig. 4 and Additional file 1), flexibility was significantly lower in the obese group than in the normal group (*P* = 0.014), and was significantly lower in the insole group than in the normal and obese groups (*P* = 0.007 and *P* = 0.014, respectively; Fig. 4). Energy absorption at application of the first axial load was significantly higher in the obese group than in the normal and insole groups (*P* = 0.007 and *P* = 0.014, respectively; Fig. 4).

**Fig. 5:**
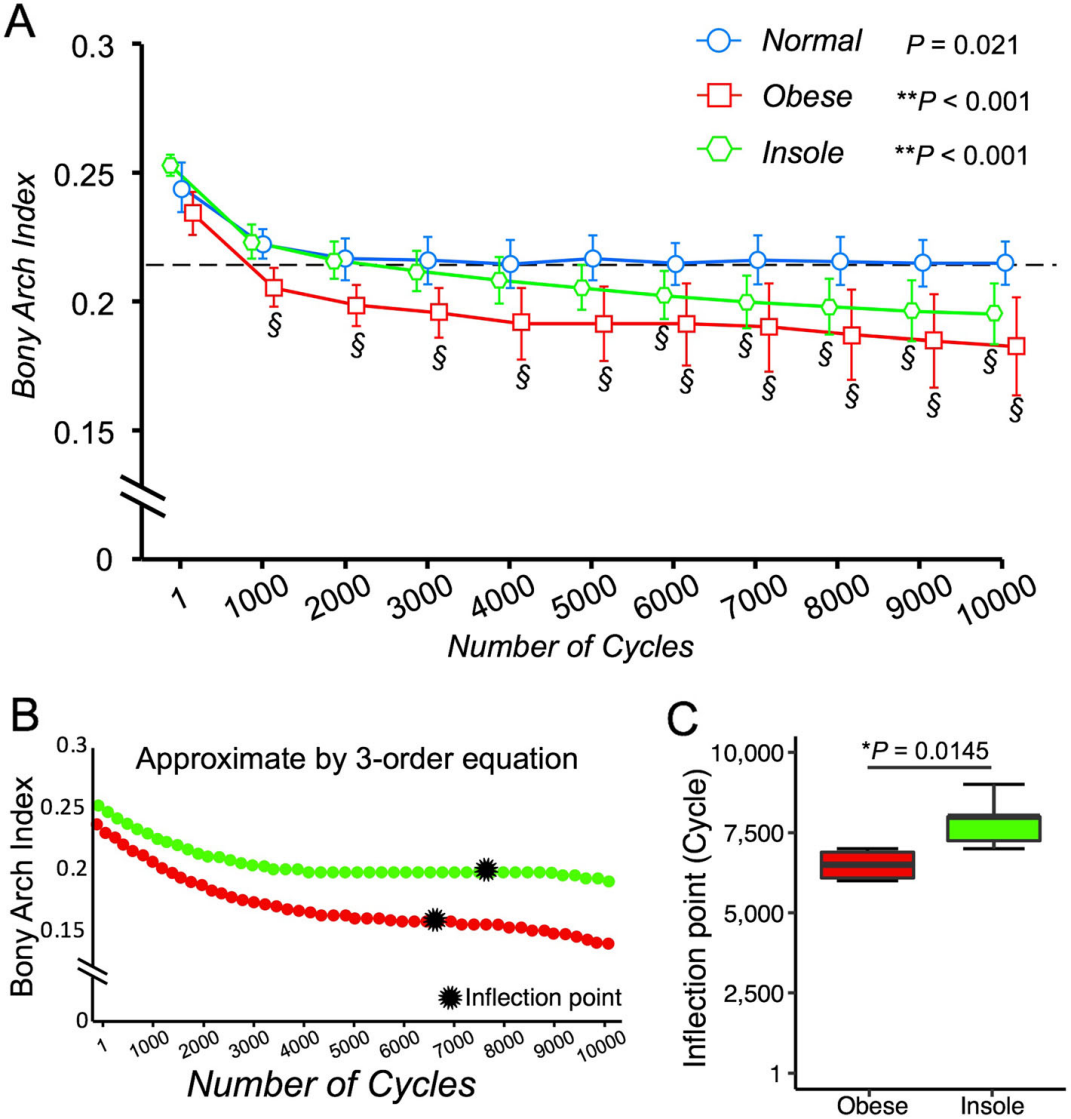
Time-dependent change in BAI in the normal, obese, and insole groups. Coordinate points represent mean change; bars represent 95% confidence intervals (**a**). BAI decreases significantly over time in all three groups (normal group, *P* = 0.0021; obese group, *P* < 0.001; insole group, *P* < 0.001)(A). The dashed line indicates the diagnostic criterion for low arch (BAI less than 0.21). § indicates BAI significantly lower than 0.21. The representive results of inflection point which indicates that the point of second to third deformity stage by approximate by 3-order eq. (**b**). The inflection point of obese group (mean, 6500 cycle; SD, 547.7) was significantly earlier than that of Insole group (mean, 7833 cycle; SD, 753) (*P* = 0.0145) (**c**)

Within-group comparisons revealed a time-dependent decrease in BAI in all three groups (normal group, *P* = 0.0021; obese group, *P* < 0.0001; insole group, *P* < 0.0001). The normal group maintained a BAI greater than 0.21 throughout the 10,000 cycles. BAI dropped significantly below 0.21 at 1000 cycles in the obese group (mean, 0.203; 95% CI, 0.196–0.209), and at 6000 cycles in the insole group (mean, 0.200; 95% CI, 0.191–0.209). The inflection point obtained from approximate by 3-order equation in obese group (mean, 6500 cycle; SD, 547.7) was significantly earlier than that of Insole group (mean, 7833 cycle; SD, 753) (*P* = 0.0145) (Fig. 5b and Fig. 5c).

**Fig. 6:**
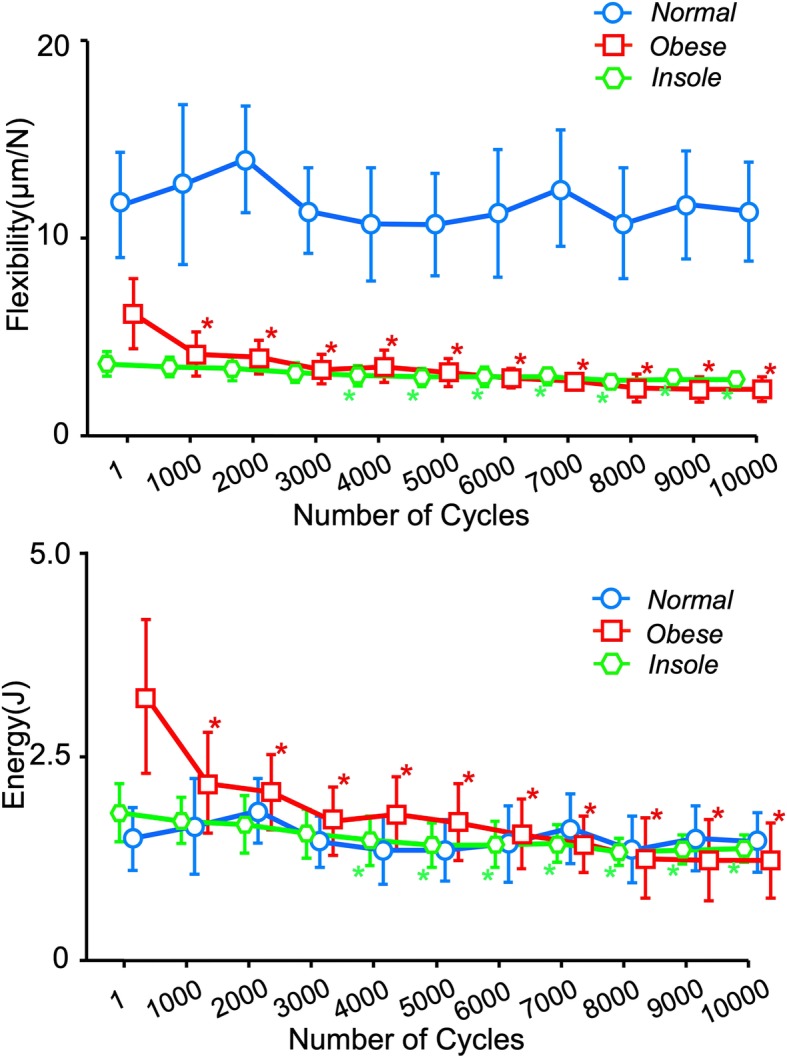
Time-dependent change in flexibility and energy absorption. Time-dependent changes in flexibility (**a**) and energy absorption (**b**) in the normal, obese, and insole groups. Coordinate points represent mean change; bars represent 95% confidence intervals. Flexibility and energy absorption decrease significantly over time in the obese and insole groups (* *P* < 0.001 for both), but not in the normal group (*P* = 0.081)

**Fig. 7:**
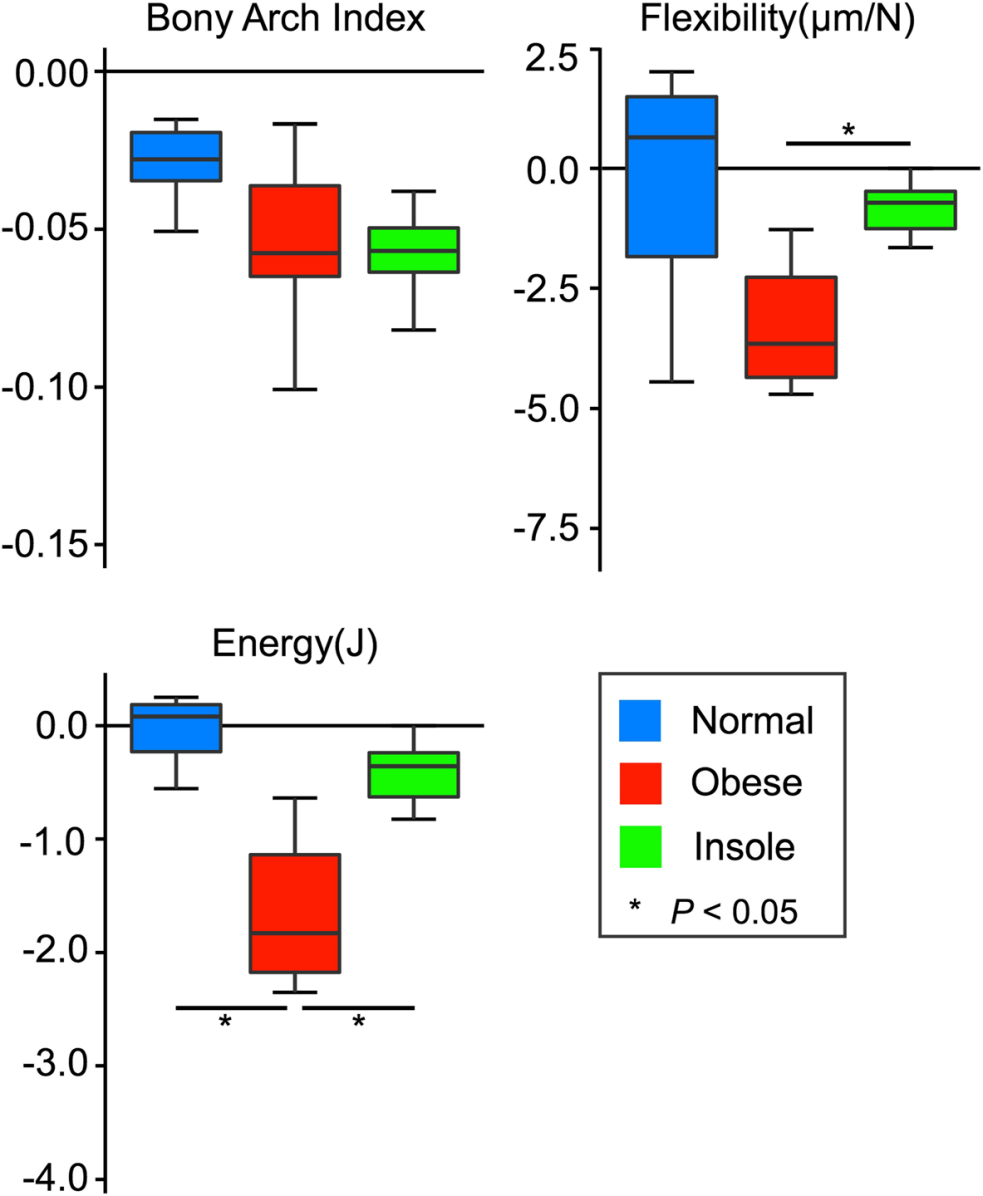
The difference between 1 and 10,000 cycles in BAI, flexibility, and energy absorption. The difference between 1 and 10,000 cycles in BAI (**a**), flexibility (**b**), and energy absorption (**c**) in the normal, obese, and insole groups. Boxes represent the interquartile range (IQR), lines within boxes represent the median, and lines outside boxes represent 1.5 times the IQR

There was no significant change in flexibility or energy absorption in the normal group (*P* = 0.081) from 1 to 10,000 cycles; however, these values decreased significantly with time in the obese and insole groups (*P* < 0.001; Fig. 6). Meanwhile, the difference between 1 and 10,000 cycles in flexibility and energy absorption in the insole group were significantly lower than in the obese group (*P* = 0.026; Fig. 7).

## Discussion

We demonstrated time-dependent changes in BAI, flexibility, and energy absorption with insole use during 10,000 cyclic loadings simulating 20,000 contiguous steps under conditions of obesity. Our results indicate that insole use could slow the progression of flatfoot in obese individuals; however, BAI, flexibility, and energy absorption of the foot may be unsustainable against 10,000 cycles of load.

It is known that insoles absorb the energy of foot loading [20] and reduce abnormal foot kinematics such as hindfoot eversion and forefoot abduction [8, 9, 21]. The increasing hindfoot eversion during weight bearing is considered to indicate abnormal foot kinematics that has the potential to increase the range of motion of the navicular bone, resulting in decreased foot rigidity and increased flexibility [22]. In this study, the insole group had lower flexibility and energy absorption than the obese group at the first load, suggesting that insoles could play a role in reducing abnormal motion and absorbing energy—a positive effect of insole function. Meanwhile, the obese group exhibited less flexibility and more energy absorption than the normal group; this suggests that the impact load and energy received in the obese group were high, as impact load increases inversely with flexibility.

Although the insole had positive effects at the first load even in the obese condition, flexibility and energy absorption decreased with time in both the obese and insole groups. Cyclic loading on bone and ligaments causes fatigue failure in the form of compressive deformation and elongation, resulting in decreased energy-absorbing capacity in these tissues [23, 24]. In the foot and ankle complex, an axial load imposes compressive force on the medial column of the foot [25] and tensile force on the plantar ligament and planter aponeurosis [26]. In this study, 10,000 cyclic loadings may have caused fatigue failure in static elements such as bones and ligaments, thus decreasing the energy-absorbing capacity of the foot. The degree of fatigue failure depends on the amount of load, and each tissue has an endurance limit which is defined as the maximum constant peak amplitude cyclic load that can be applied in an infinite number of stress cycles without causing failure [17]. In the foot and ankle, increasing loads on the foot cause excess eversion of the hindfoot [11, 12], which increases tensile force on the spring ligament and plantar ligament [26]. In this study, higher cyclic loads on the foot appeared to exceed the endurance limit in both the obese and insole groups, but not in the normal group.

Dynamic elements such as muscle contraction force are another factor that influences energy absorption in the foot [14, 27]. In particular, the posterior tibialis muscle is important for maintaining arch height, and reduces the energy imposed on the foot and ankle [28]. Anatomically, the posterior tibialis tendon turns sharply around the medial malleolus towards the navicular bone, generating gliding resistance around the medial malleolus [29]. This resistance would be higher when the foot arch is flattened and the arc of contact of the posterior tibialis tendon is increased [29]. BAI fell below 0.21 in both the obese group and the insole group in this study, indicating that increased gliding resistance in flatfoot may reduce the force transmitted by the posterior tibialis tendon. Hirano et al. reported that an insole did not reduce the gliding resistance of the posterior tibial tendon, even when correct foot alignment was achieved [30]. Thus, the insole in our study may not have positively affected dynamic elements during cyclic loading.

In our experimental model, BAI decreased significantly over time in all three groups. Cyclic loading causes fatigue behavior, which is similar to creep behavior [18]; the deformation rate is relatively high in the initial stage, is relatively constant and slow in the second stage, and reaccelerates in the third stage [18]. As our experimental system reproduces physiological structural change, the marked decrease in BAI from 1 to 1000 cycles in all three groups may represent the initial stage of deformation. Furthermore, the time-dependent decreases in flexibility and BAI in the obese group may represent stage III AAFD with foot rigidity [31].

This study has several limitations. First, we demonstrated the time-dependent effect of insole use with a cyclic loading-displacement measurement device, which is limited to 2-dimensional foot kinematics. Because AAFD patients show multifactorial changes [32], further studies should evaluate 3-dimensional foot deformation. Second, our experimental system only simulated the midstance phase of gait. A previous biomechanical study using dynamic gait simulator showed that the dorsiflexion and eversion were increased during the terminal stance phase in the calcaneus and first metatarsal bone, respectively, and the range of motion of the calcaneus and the first metatarsal bone was increased in the flatfoot model [33]. Although these terminal stance phases involve different biomechanical stresses with the midstance phase, our results only showed that the silicone rubber insole could help absorb energy and increase foot stability at the midstance phase of gait. It is important to analysis the effect of the insole, especially the effect of metatarsal support, during the terminal stance phase of gait. Third, only one type of insole was tested in this study. Although the shape and material of the tested insole are typical of commercial products, other shapes and materials should be evaluated to further elucidate the effects of insole function. Fourth, specimens were randomly assigned to the three groups, and axial load and tendon traction were applied without regard to the body weight of the cadaver. In living subjects, obesity is known to cause muscle and bone weakness [34] and tendon and ligament fragility [32]; therefore, collapse of the foot arch in obese individuals may occur at an earlier stage than is suggested by our results. Fifth, the flexibility and energy absorption of the foot in this study was calculated as that in an elastic and homogeneous body; therefore, it might be slightly different from the actual value because the foot and ankle are complicated structures that include the joints and a variety of different tissues. Sixth, only the posterior tibialis muscle was activated in this study. In their cadaveric study, Blackman et al. demonstrated that overactivation of the Achilles tendon causes abnormal motion of the calcaneus, leading to AAFD progression [35]. Although activation of the Achilles tendon or other extrinsic or intrinsic muscles of the foot may promote a change in arch height, activation of the posterior tibialis tendon maintained normal arch height under normal-weight conditions in our study, even after 10,000 loading cycles [14]. On the basis of our results, we conclude that posterior tibialis function is involved in AAFD; however, further study is necessary to better understand the coordinated muscle function on the foot arch.

## Conclusion

Use of insoles by obese individuals may help absorb energy and increase foot stability. These positive effects of insole function serve to slow time-dependent kinetic changes; however, the effect was not enough to maintain BAI against 6000 hyper-load cycles in this study. The positive effects of insole for obese individuals might be limited by number of steps.

## Supplementary information

**Additional file 1.** The BAI for non-weight bearing before the first load was not different significantly among normal, obese and insole groups. Quantitative data for each specimen are shown in mean ± SE with dot plots.

## Data Availability

Data and materials not indicated in this manuscript are available from the corresponding author.

## Abbreviations

AAFD: Adult acquired flatfoot deformity
ADL: Activities of daily living
BAI: Bony arch index
BMI: Body mass index
CCD: Charge-coupled device
LED: Light-emitting diode

## References

[1] Walters JL, Mendicino SS (2014). The flexible adult flatfoot: anatomy and pathomechanics. Clin Podiatr Med Surg.

[2] Pinney SJ, Lin SS (2006). Current concept review: acquired adult flatfoot deformity. Foot Ankle Int..

[3] Tenenbaum S, Hershkovich O, Gordon B, Bruck N, Thein R, Derazne E (2013). Flexible pes planus in adolescents: body mass index, body height, and gender--an epidemiological study. Foot Ankle Int. SAGE Publications.

[4] Nielsen MD, Dodson EE, Shadrick DL, Catanzariti AR, Mendicino RW, Malay DS (2011). Nonoperative care for the treatment of adult-acquired flatfoot deformity. J Foot Ankle Surg.

[5] Elattar O, Smith T, Ferguson A, Farber D, Wapner K. Uses of Braces and Orthotics for Conservative Management of Foot and Ankle Disorders. Foot & Ankle Orthopaedics. 9 ed. 2018;3:247301141878070–12. 10.1177/24730114231193419 PMC10408344 37566687

[6] Chao W, Wapner KL, Lee TH, Adams J, Hecht PJ (1996). Nonoperative management of posterior tibial tendon dysfunction. Foot Ankle Int.

[7] Toullec E (2015). Adult flatfoot. Orthop Traumatol Surg Res.

[8] Kitaoka Harold B., Luo Zong Ping, An Kai-Nan (1997). Analysis of Longitudinal Arch Supports in Stabilizing the Arch of the Foot. Clinical Orthopaedics and Related Research.

[9] Kitaoka HB, Luo ZP, Kura H, An KN (2002). Effect of foot orthoses on 3-dimensional kinematics of flatfoot: a cadaveric study. Arch Phys Med Rehabil.

[10] Augustin JF, Lin SS, Berberian WS, Johnson JE (2003). Nonoperative treatment of adult acquired flat foot with the Arizona brace. Foot Ankle Clin.

[11] Huang CK, Kitaoka HB, An KN, Chao EY (1993). Biomechanical evaluation of longitudinal arch stability. Foot Ankle.

[12] Tochigi Y (2003). Effect of arch supports on ankle-subtalar complex instability: a biomechanical experimental study. Foot Ankle Int..

[13] Cowan DN, Jones BH, Robinson JR (1993). Foot morphologic characteristics and risk of exercise-related injury. Arch Fam Med.

[14] Kamiya T, Uchiyama E, Watanabe K, Suzuki D, Fujimiya M, Yamashita T (2012). Dynamic effect of the tibialis posterior muscle on the arch of the foot during cyclic axial loading. Clin Biomech (Bristol, Avon)..

[15] Perry J, Burnfield JM. Gait Analysis Slack. 2010.

[16] Silver RL, la Garza de J, Rang M (1985). The myth of muscle balance. A study of relative strengths and excursions of normal muscles about the foot and ankle. J Bone Joint Surg Br.

[17] Nordin M, Frankel VH. Basic biomechanics of the musculoskeletal system: Lippincott Williams & Wilkins; 2001.

[18] Wren TAL, Lindsey DP, Beaupré GS, Carter DR (2003). Effects of creep and cyclic loading on the mechanical properties and failure of human Achilles tendons. Ann Biomed Eng.

[19] Kanda Y (2013). Investigation of the freely available easy-to-use software “EZR” for medical statistics. Bone Marrow Transplant.

[20] Chiu H-T, Shiang T-Y (2007). Effects of insoles and additional shock absorption foam on the cushioning properties of sport shoes. J Appl Biomech.

[21] Neville C, Flemister AS, Houck JR (2009). Effects of the AirLift PTTD brace on foot kinematics in subjects with stage II posterior tibial tendon dysfunction. J Orthop Sports Phys Ther.

[22] Blackwood CB, Yuen TJ, Sangeorzan BJ, Ledoux WR (2005). The midtarsal joint locking mechanism. Foot Ankle Int..

[23] Carter DR, Caler WE, Spengler DM, Frankel VH (1981). Fatigue behavior of adult cortical bone: the influence of mean strain and strain range. Acta Orthop Scand.

[24] Woo SL, Debski RE, Zeminski J, Abramowitch SD, Saw SS, Fenwick JA (2000). Injury and repair of ligaments and tendons. Annu Rev Biomed Eng.

[25] MANTER JT (1946). Distribution of compression forces in the joints of the human foot. Anat Rec.

[26] Kelikian AS, Sarrafian SK. Sarrafian's anatomy of the foot and ankle: Lippincott Williams & Wilkins; 2011.

[27] Kitaoka HB, Luo ZP, An KN (1997). Effect of the posterior Tibial tendon on the arch of the foot during simulated Weightbearing: biomechanical analysis. Foot Ankle Int SAGE Publications.

[28] Kokubo T, Hashimoto T, Nagura T, Nakamura T, Suda Y, Matsumoto H (2012). Effect of the posterior Tibial and peroneal longus on the mechanical properties of the foot arch. Foot Ankle Int SAGE Publications.

[29] Uchiyama E, Kitaoka HB, Fujii T, Luo ZP, Momose T, Berglund LJ (2006). Gliding resistance of the posterior tibial tendon. Foot Ankle Int..

[30] Hirano T, McCullough MBA, Kitaoka HB, Ikoma K, Kaufman KR (2009). Effects of foot orthoses on the work of friction of the posterior tibial tendon. Clin Biomech (Bristol, Avon).

[31] Haddad SL, Myerson MS, Younger A, Anderson RB, Davis WH, Symposium MA (2011). Adult acquired flatfoot deformity. Foot Ankle Int..

[32] Zhang Y, Xu J, Wang X, Huang J (2013). An in vivo study of hindfoot 3D kinetics in stage II posterior tibial tendon dysfunction (PTTD) flatfoot based on weight-bearing CT scan. Bone and Joint ….

[33] Watanabe K, Kitaoka HB, Fujii T, Crevoisier X, Berglund LJ, Zhao KD (2013). Posterior tibial tendon dysfunction and flatfoot: analysis with simulated walking. Gait & posture.

[34] Ilich JZ, Kelly OJ, Inglis JE, Panton LB, Duque G, Ormsbee MJ (2014). Interrelationship among muscle, fat, and bone: connecting the dots on cellular, hormonal, and whole body levels. Ageing Res Rev.

[35] Blackman AJ, Blevins JJ, Sangeorzan BJ, Ledoux WR (2009). Cadaveric flatfoot model: ligament attenuation and Achilles tendon overpull. J Orthop Res.

