# Supplementary figures and images for: Can an insole for obese individuals maintain the arch of the foot against repeated hyper loading?
Source: BMC Musculoskelet Disord. 2019 Oct 11;20:442. doi: 10.1186/s12891-019-2819-2 (PMC6790017; doi:10.1186/s12891-019-2819-2)

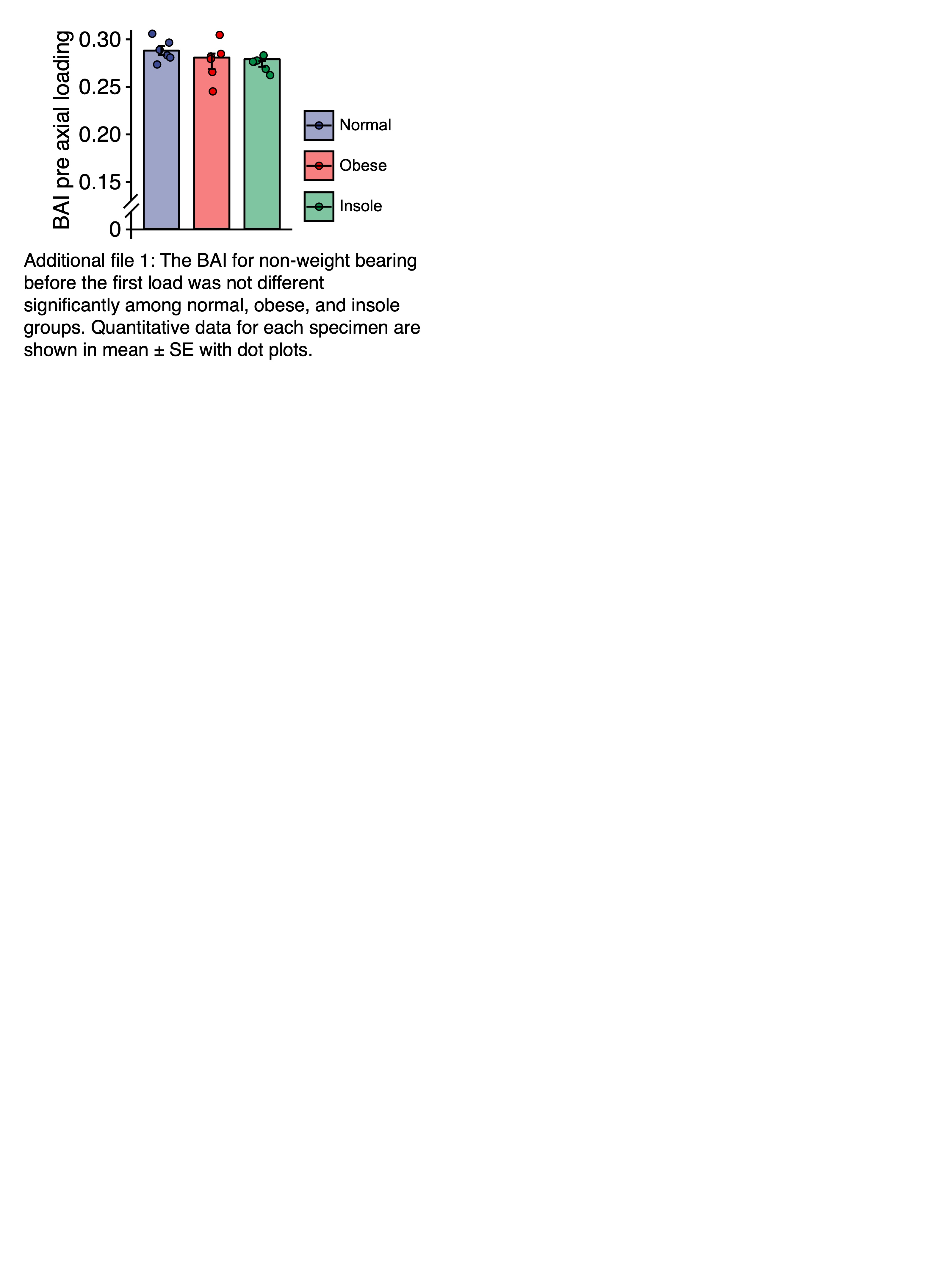

Supplement: Supplementary file 1 — Additional file 1. The BAI for non-weight bearing before the first load was not different significantly among normal, obese and insole groups. Quantitative data for each specimen are shown in mean ± SE with dot plots. [file 12891_2019_2819_MOESM1_ESM.jpg]
